# Oligomerization of the *E. coli* Core RNA Polymerase: Formation of (α_2_ββ'ω)_2_–DNA Complexes and Regulation of the Oligomerization by Auxiliary Subunits

**DOI:** 10.1371/journal.pone.0018990

**Published:** 2011-04-20

**Authors:** Seema G. Kansara, Maxim V. Sukhodolets

**Affiliations:** Department of Chemistry and Biochemistry, Lamar University, Beaumont, Texas, United States of America; New England Biolabs, Inc., United States of America

## Abstract

In this work, using multiple, dissimilar physico-chemical techniques, we demonstrate that the *Escherichia coli* RNA polymerase core enzyme obtained through a classic purification procedure forms stable (α_2_ββ'ω)_2_ complexes in the presence or absence of short DNA probes. Multiple control experiments indicate that this self-association is unlikely to be mediated by RNA polymerase-associated non-protein molecules. We show that the formation of (α_2_ββ'ω)_2_ complexes is subject to regulation by known RNA polymerase interactors, such as the auxiliary SWI/SNF subunit of RNA polymerase RapA, as well as NusA and σ^70^. We also demonstrate that the separation of the core RNA polymerase and RNA polymerase holoenzyme species during Mono Q chromatography is likely due to oligomerization of the core enzyme. We have analyzed the oligomeric state of the polymerase in the presence or absence of DNA, an aspect that was missing from previous studies. Importantly, our work demonstrates that RNA polymerase oligomerization is compatible with DNA binding. Through *in vitro* transcription and *in vivo* experiments (utilizing a RapA^R599/Q602^ mutant lacking transcription-stimulatory function), we demonstrate that the formation of tandem (α_2_ββ'ω)_2_–DNA complexes is likely functionally significant and beneficial for the transcriptional activity of the polymerase. Taken together, our findings suggest a novel structural aspect of the *E. coli* elongation complex. We hypothesize that transcription by tandem RNA polymerase complexes initiated at hypothetical bidirectional “origins of transcription” may explain recurring switches of the direction of transcription in bacterial genomes.

## Introduction

In *Escherichia coli*, the chromosomal DNA is replicated by the DNA polymerase III complex, which contains two copies of the pol III core connected through a single clamp loader complex [1], [2]. It is thought that a single-copy core RNA polymerase complex consisting of α_2_ββ'ω subunits transcribes the same DNA [3]. It appears that the necessity of simultaneous amplification of complementary strands of double-stranded DNA can justify the existence of tandem replication complexes in the case of DNA polymerase; this rationale is lacking in the case of RNA polymerase, which utilizes only one DNA strand as a template.

When the *E. coli* RNA polymerase was initially isolated and characterized, multiple, independent studies reported self-association of RNA polymerase *in vitro* [4]–[8]. However, there was no accord with respect to the exact oligomeric states of the observed complexes. In different studies, the formation of tetrameric, hexameric, and octameric RNA polymerase complexes was suggested [7]–[9]. The question of whether DNA-bound RNA polymerase can self-associate (which is important for understanding the possible functional implications of such self-association), to the best of our knowledge, has not been addressed in any of the existing studies.

In this work, using multiple, dissimilar physico-chemical methods, we demonstrate that the *E. coli* RNA polymerase core enzyme, obtained through a purification procedure similar to that developed by Hager *et al.* [10], forms stable (α_2_ββ'ω)_2_ complexes in the presence or absence of short DNA probes. Multiple control experiments indicate that this association is unlikely to be mediated by accessory nucleic acids or other RNA polymerase-associated non-protein molecules. Furthermore, we show that the formation of these complexes is subject to regulation by RapA, NusA, and σ^70^. Our data demonstrate a physical interaction between individual α_2_ββ'ω complexes *in vitro* and the likely functional significance of this association for the transcriptional activity of the polymerase. We have also analyzed the oligomeric state of the polymerase in the presence or absence of DNA, an element that was missing from previous *E. coli* RNA polymerase studies. Our findings suggest a novel structural aspect of the *E. coli* elongation complex that warrants further studies addressing the functional significance of *E. coli* RNA polymerase self-association *in vivo*.

## Results

**Figure 1:**
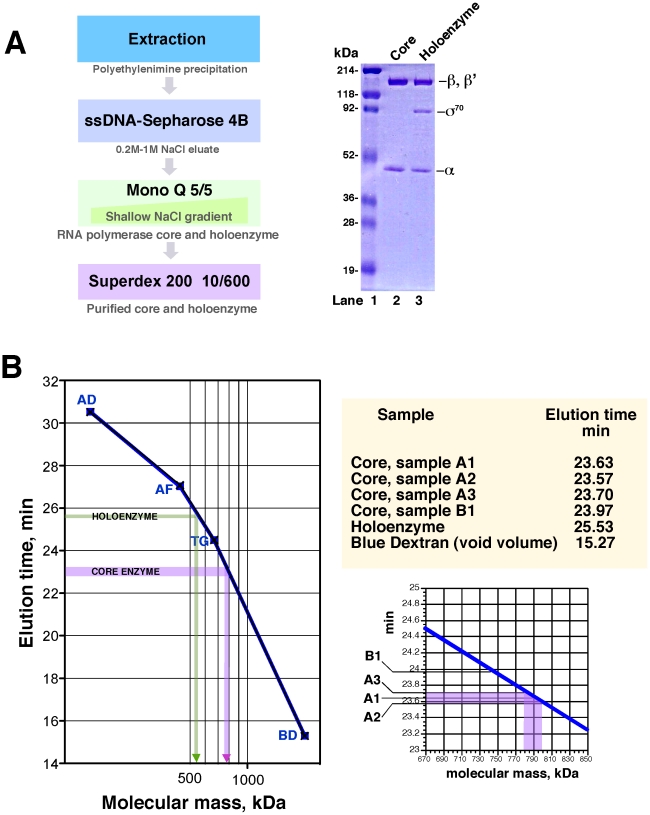
Determination of the molecular masses of the core RNA polymerase and the RNA polymerase holoenzyme under non-denaturing conditions. **A.** Schematic for the RNA polymerase purification procedure. Samples of the purified core RNA polymerase (lane 2) and the RNA polymerase holoenzyme (lane 3) (∼4 µg of each complex) are shown on a Coomassie-stained SDS gel. The core RNA polymerase and the RNA polymerase holoenzyme were purified as described in Materials and Methods. **B.** Determination of the molecular masses of the core RNA polymerase and the RNA polymerase holoenzyme by gel-permeation chromatography on a Superose 6 HR 10/300 column. Chromatography was carried out as described in Materials and Methods. Native protein markers (Sigma) were: Alcohol Dehydrogenase (AD, 150 kDa), Apoferritin (AF, 443 kDa), Thyroglobulin (TG, 669 kDa), and Blue Dextran (BD, 2000 kDa). Each marker was run twice (black crosses and blue rectangles). Elution times for the RNA polymerase samples are indicated.

Due to our interest in the function and mechanism of the RNA polymerase-associated SWI/SNF homolog RapA [11]–[13], we purify *Escherichia coli* RNA polymerase on a regular basis utilizing a protocol reminiscent of that developed by Hager *et al*. [10] (Figure 1A). In our early studies with RapA, we observed that the core RNA polymerase eluted before the RNA polymerase holoenzyme during gel-permeation chromatography [14] (implying dissimilar oligomeric states for the two forms of the polymerase). However, the apparent molecular masses of these RNA polymerase complexes were not determined at that time. We re-examined the molecular masses of these RNA polymerase complexes during the course of our recent work. In accord with our previously reported data, at 0.1 M NaCl, the *E. coli* core RNA polymerase eluted before the RNA polymerase holoenzyme. It ran as a single peak with an apparent molecular mass of 780–800 kDa, which is approximately double the predicted molecular mass of an RNA polymerase core enzyme ‘monomer’ and well within the column's separation range (>8 min after the column's void volume) (Figure 1B). The mobility of the RNA polymerase holoezyme in these experiments was consistent with its known molecular mass (Figure 1B, green arrow). Because the ratios of the alpha and beta subunits in the observed 780–800 kDa core enzyme species were identical to those in the input material (judging from PAGE analysis; data not shown), we identified this enzyme species as (α_2_ββ'ω)_2_ core RNA polymerase complexes.

We questioned whether the core RNA polymerase purified in this manner could have retained small accessory molecules – such as nucleic acid fragments or lipids – which could have led to the self-association of individual α_2_ββ'ω complexes. To remove such (hypothetical) accessory material, we subjected core enzyme from the final purification step in the protocol described in Figure 1A to multiple rounds of >20-fold dilution with TGED buffer containing 0.5 M NaCl, followed by concentration to the original volume in centrifugal ultrafiltration devices with a 30-kDa cutoff. The mobility of the resulting core enzyme samples during gel-filtration was similar to that of the input material (Figure 1B, right panels, sample B1). We also attempted to detect accessory nucleic acids in the core enzyme preparations by T4 PNK labeling in the presence of [γ^32^P] ATP. Likewise, these experiments showed no detectable radioactivity co-migrating with the core enzyme in gel-filtration (data not shown).

**Figure 2:**
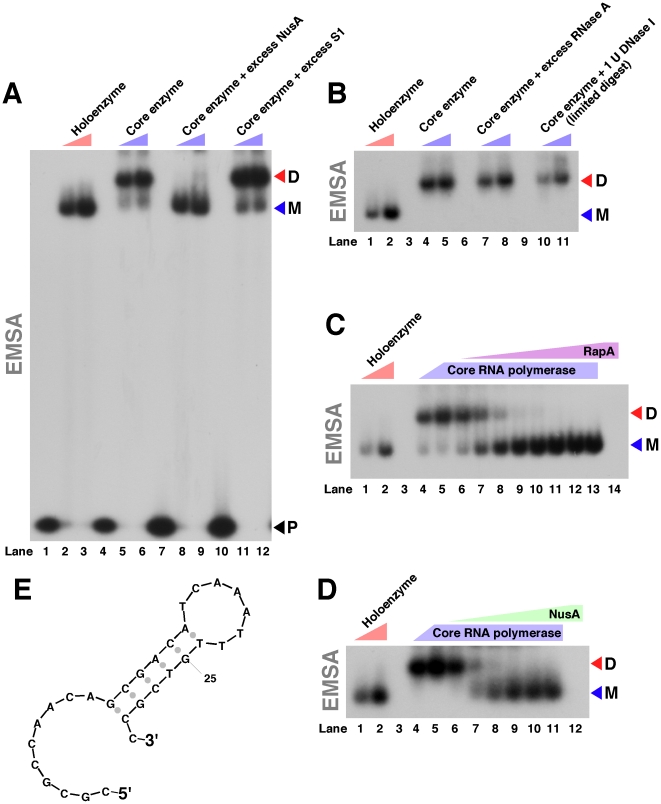
Core RNA polymerase–DNA and RNA polymerase holoenzyme–DNA complexes show dissimilar mobilities in native PAGE. EMSA experiments were carried out as described in Materials and Methods. M: single-copy RNA polymerase–DNA complexes; D: (α_2_ββ'ω)_2_–DNA complexes; P: free DNA probe. **A.** Lanes 1 and 4: DNA probe alone. Lanes 2 and 3: DNA plus 80 or 160 nM purified RNA polymerase holoenzyme, respectively. Lanes 5 and 6: DNA plus 80 or 160 nM purified core RNA polymerase, respectively. Lane 7: DNA plus 200 nM purified NusA. Lanes 8 and 9: as lanes 5 and 6, but with 200 nM purified NusA. Lane 10: DNA plus 200 nM purified S1. Lanes 11 and 12: as lanes 5 and 6, but with 200 nM purified S1. **B.** Lanes 1 and 2: DNA plus 40 or 160 nM purified RNA polymerase holoenzyme, respectively. Lanes 4 and 5: DNA plus 40 or 160 nM purified core RNA polymerase, respectively. Lanes 7 and 8: as lanes 4 and 5, but with excess (4 U) RNase A. Lanes 10 and 11: as lanes 4 and 5, but with 1 U DNase I. **C.** and **D.** Lanes 1 and 2 in both gels contained DNA plus 40 or 160 nM purified RNA polymerase holoenzyme, respectively. Lanes 4 and 5 in both gels contained DNA and 40 or 160 nM purified core RNA polymerase, respectively. Lanes 6–13 in the gel shown in Figure 2C contained DNA plus 160 nM purified core RNA polymerase and increasing amounts of purified RapA. Lanes 6–11 in Figure 2D contained DNA plus 160 nM purified core RNA polymerase and increasing amounts of purified NusA. See text for additional details. In an independent set of experiments we confirmed that titration of the core enzyme with purified NusA results in the appearance of a distinct core ‘monomer’–NusA complex during native PAGE as revealed by Coomassie blue staining (Supporting Information, Figure S1; therefore the observed mobility shift of the ^32^P-labeled DNA is unlikely due to a mere redistribution of the ^32^P-labeled DNA probe). Note that minor mobility differences between single-copy core and holoenzyme species could be observed on lower porosity polyacrylamide gels, as seen in Figure 3 below. **E.** Schematic of the DNA probe used in the binding experiments described above.

Next, we sought (a) an alternative technique not relying on gel-permeation chromatography to compare the molecular masses of the core RNA polymerase and the RNA polymerase holoenzyme under non-denaturing conditions and (b) to test the effect of DNA on the stability of the observed core RNA polymerase complexes. For this purpose, we used a short (30-nt), ^32^P 5′ end-labeled DNA probe with the indicated secondary structure (Figure 2E) and compared the mobilities of the core RNA polymerase–DNA and RNA polymerase holoenzyme–DNA complexes during native PAGE. Consistent with the results of the gel-permeation chromatography experiments, the core RNA polymerase and the RNA polymerase holoenzyme showed dissimilar electrophoretic mobilities (Figure 2A). There was a clear separation between the core RNA polymerase–DNA and RNA polymerase holoenzyme–DNA complexes; the core enzyme migrated more slowly than the RNA polymerase holoenzyme (Figure 2). These results were consistent with the results of our gel-filtration studies. Excess Ribonuclease A and a (limited) DNase I digest failed to alter the mobility of the core RNA polymerase-DNA complexes (Figure 2B), suggesting that the apparent association of the core RNA polymerase molecules was not mediated by accessory nucleic acid.

Next, we tested whether interaction with RapA, an auxiliary SWI/SNF subunit of *E. coli* RNA polymerase [11]–[15], could affect the stability of (α_2_ββ'ω)_2_ complexes. We observed a significant shift in the core enzyme's electrophoretic mobility in the presence of RapA, which was consistent with its conversion to the ‘monomeric’ state in the presence of RapA (Figure 2C). The single-copy configuration of the core RNA polymerase–RapA–DNA complexes was apparent due to their co-migration with RNA polymerase holoenzyme–DNA complexes possessing a comparable molecular mass (Figure 2C, compare lanes 1–2 to lanes 7–13). The RNA polymerase holoenzyme showed no tendency to form oligomers during native PAGE, in accord with the results of our gel-filtration-based studies. Importantly, this set of experiments demonstrated the existence of two distinct forms of the core enzyme with dissimilar electrophoretic mobilities in native PAGE (Figure 2C, lanes 6–8). Another key interactor of *E. coli* RNA polymerase, the NusA protein – which is described in the existing literature as an elongation factor and a co-factor of antiterminators [16]–[19] – also dissociated the putative tandem core enzyme–DNA complexes (Figure 2D). Excess S1 and Hfq (the former protein being a potential low-affinity interactor of RNA polymerase [20]) – used as controls – did not alter the electrophoretic mobility of the core RNA polymerase–DNA complexes (Figure 2A, lanes 11 and 12, and data not shown).

**Figure 3:**
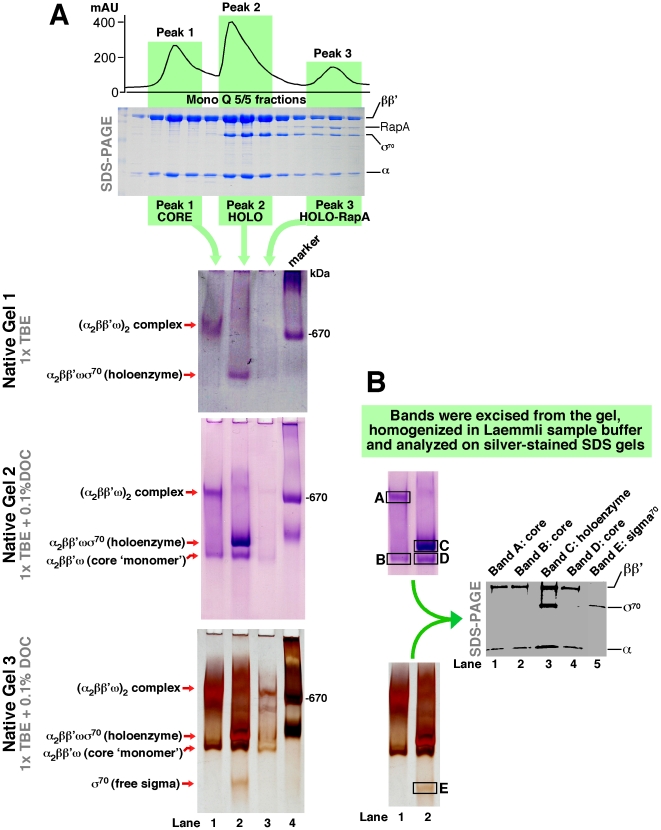
Formation of putative (α_2_ββ'ω)_2_ complexes by the *E. coli* core RNA polymerase. **A.** *E. coli* RNA polymerase was isolated as described [11], [20], [24] up to the Mono Q stage, and the content of individual Mono Q peaks (containing the core RNA polymerase, RNA polymerase holoenzyme, and the holoenzyme–RapA complex [11]) was analyzed by non-denaturing electrophoresis on 5% polyacrylamide gels in the absence (Gel 1) or in the presence of DOC (Gels 2 and 3). The electrophoresis buffers for Gels 1–3 are indicated in the figure legend. Lanes 1–3 of each gel contained approximately 20 µl of the Mono Q material. Gels 1 and 2 were stained with Imperial Purple™ (Pierce) (a colloidal Coomassie-based stain). Gel 3 was stained with silver, which enabled visualization of free σ^70^ subunit, a small fraction of which dissociates from the holoenzyme in the presence of DOC. Note the resulting single-copy core and holoenzyme complexes in lane 2 of Gels 2 and 3. The Mono Q A_280_ profile and the Mono Q fractions analyzed by SDS PAGE (10 µl from each fraction/lane) are shown at the top. The RNA polymerase subunits are indicated. **B.** To confirm the identity of individual complexes and/or proteins (indicated schematically at the left in Figure 3A), the Coomassie-stained bands highlighted by rectangle frames in Gel 2 were excised (from the actual gel shown here) and homogenized in ∼200 µl of Laemmli sample buffer in Eppendorf tube-size disposable homogenizers. Following precipitation of the polyacrylamide slurry by centrifugation, the supernatants were analyzed on silver-stained SDS gels (shown at the right).

Because Mono Q chromatography also efficiently separates the core RNA polymerase and the RNA polymerase holoenzyme ([10] and [11]; a somewhat surprising result given the overall similarity of the two complexes), we further analyzed the Mono Q-purified RNA polymerase complexes (that is, the core RNA polymerase, RNA polymerase holoenzyme, and the holoenzyme–RapA complex [11]) by native, non-denaturing electrophoresis (Figure 3). In 1× TBE gels (in the absence of detergents and/or chaotropic agents), the core RNA polymerase and the RNA polymerase holoenzyme showed distinctly different mobilities (Figure 3A, lanes 1 and 2 in Gel 1). These results are in accord with (a) our gel filtration-based studies and (b) our native PAGE-based analysis of the same complexes in the presence of ^32^P-labeled DNA probes. To rule out the possibility that the ‘atypical’ mobility of the core enzyme was the result of conformational changes (as opposed to tandem complex formation), we again sought evidence for the existence of two distinct forms of the core enzyme in the absence of DNA. To this end, we analyzed the content of three key Mono Q peaks by native PAGE in the presence of various detergents. This set of experiments also tested potential contributions of small RNA polymerase-associated hydrophobic molecules (such as lipids) to RNA polymerase oligomerization. Excess deoxycholate (DOC) (the detergent used during the extraction stage in the RNA polymerase purification procedure) did not preclude the formation of (α_2_ββ'ω)_2_ complexes (Figure 3A, gels 2 and 3); however, DOC shifted the equilibrium between α_2_ββ'ω and (α_2_ββ'ω)_2_ forms toward single-copy complexes (Figure 3A). The formation of both types of complexes during native PAGE in the presence of 0.1% DOC was convincing (Figures 3A and 3B). Note that the identity/subunit composition of the observed complexes was further confirmed by SDS-PAGE analysis of individual protein complexes excised from native polyacrylamide gels (Figure 3B). No higher-order oligomers were detected (Figure 3A, lane 1 in Gels 1–3), thus arguing against ‘head-to-tail’ association of individual RNA polymerase molecules. The results of this set of experiments (carried out with marginally diluted but otherwise unadulterated Mono Q fractions) strongly imply that efficient separation of the peaks containing the ‘core RNA polymerase’ and the ‘RNA polymerase holoenzyme’ (as judged by SDS-PAGE, Figure 3A, top) during Mono Q chromatography is due to the distinctly different oligomeric states of the two forms of RNA polymerase, rather than to core enzyme–σ^70^ piggyback interaction alone.

**Figure 4:**
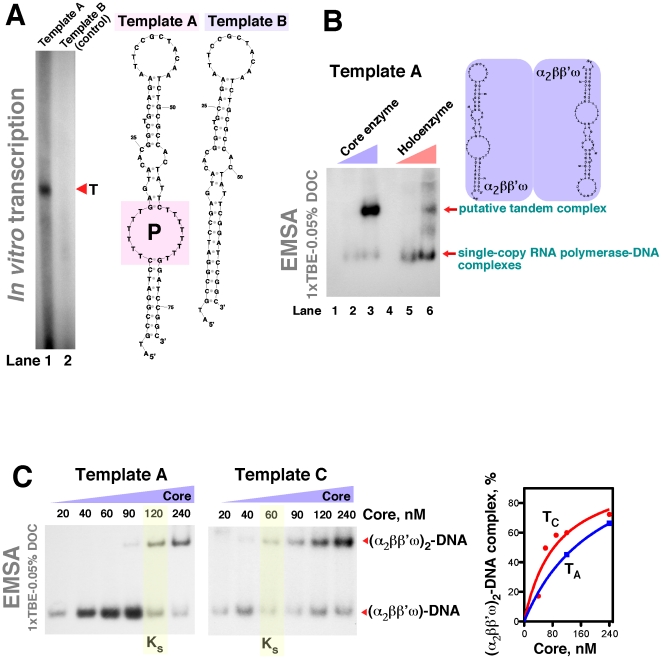
Formation of putative tandem complexes by the DNA-bound *E. coli* RNA polymerase. **A.** Transcriptional activity of the *E. coli* RNA polymerase with a synthetic hairpin-shaped DNA template incorporating an asymmetric dT-mismatch bubble (P). Note the production of a template-specific transcript (T) for Template A and little or no transcriptional activity with the control template (Template B) (both templates are shown schematically at the right). *In vitro* transcription was carried out in buffer D, as described in Materials and Methods. **B.** Formation of the (α_2_ββ'ω)_2_–Template A complexes. Reactions in lanes 2–3 and 5–6 were similar to those, respectively, in lanes 4–5 and 1–2 in Figure 2B, except that ^32^P 5′ end-labeled Template A DNA was used in this set, and the gel contained 0.05% DOC to visualize both single-copy RNA polymerase–DNA and (α_2_ββ'ω)_2_–DNA complexes. **C.** Similar to Figure 4B, but with a more gradual incremental increase in the core enzyme concentrations. Template C was similar to Template A, except that it contained a (dT)_10_ extension at the 5′ end. Quantitated results of the experiments are shown in the panel at the right. T_A_: Template A; T_C_: Template C.

Next, we sought to demonstrate that the formation of (α_2_ββ'ω)_2_ complexes is compatible with the presence of duplex DNA templates. To this end, we took advantage of a set of extra-short – yet transcriptionally active – DNA templates that were recently constructed in connection with our study of the function and catalytic mechanism of RapA. In the hairpin-shaped DNA template selected for this analysis, shown schematically in Figure 4A (Template A), transcription initiation is triggered by an asymmetric dT-mismatch bubble. Removal of this structure (Template B) abolishes the production of transcripts from this template by RNA polymerase (Figure 4A, gel, compare lanes 1 and 2). This family of DNA templates (the lengths of which are comparable with *E. coli* RNA polymerase DNA footprints) also supported the formation of (α_2_ββ'ω)_2_–DNA transcription complexes (Figures 4B and 4C) – in accord with the results of the experiments with shorter DNA probes described in Figure 2.

**Figure 5 pone-0018990-g005:**
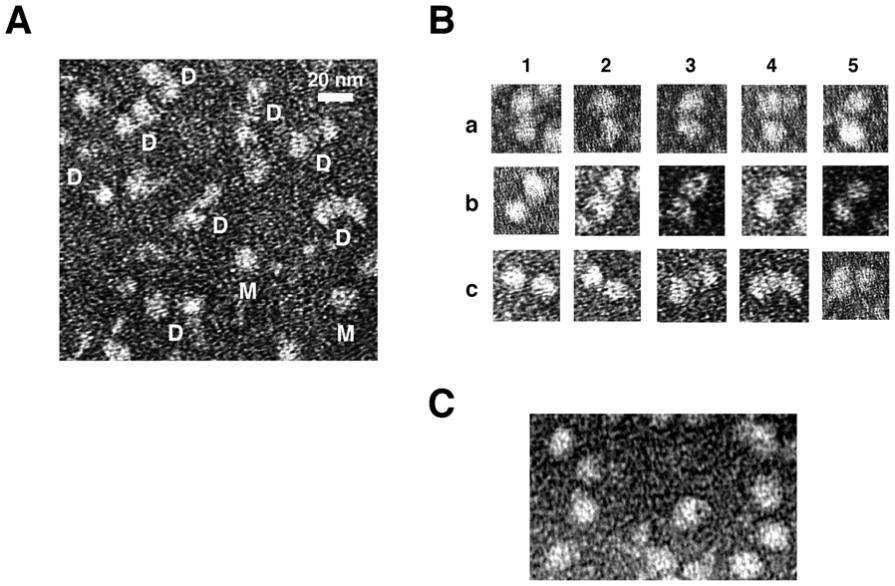
Electron-microscopic analysis of the oligomeric structure of the *E. coli* RNA polymerase core enzyme. Electron microscopy was carried out as previously described [23]. In panel **A**, ‘dimeric’ (D) and ‘monomeric’ RNA polymerase complexes (M) are indicated. Characteristic types of (α_2_ββ'ω)_2_ complexes are shown in panel **B**; control images of the RNA polymerase holoenzyme are shown in panel **C**.

Consistent with the gel-exclusion chromatography- and nondenaturing PAGE-based studies, electron microscopy revealed distinct ‘dimeric’ complexes in core RNA polymerase preparations (Figures 5A and 5B). Both ‘dimeric’ and ‘monomeric’ forms of the core enzyme were typically observed, with the former being the predominant species (Figure 5A).

**Figure 6 pone-0018990-g006:**
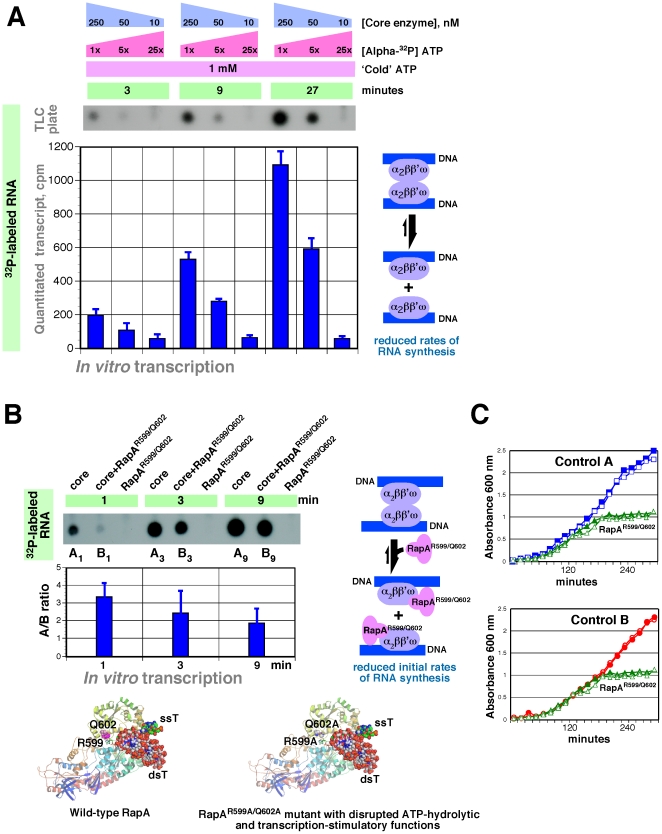
Effect of oligomerization on transcriptional activity of RNA polymerase. **A.** Concentration-dependent ‘activation’ of the *E. coli* RNA polymerase. *In vitro* transcription reactions were carried out in buffer B (see Materials and Methods) with a Poly (dA)^.^Poly(dT) DNA template (GE Healthcare); the concentrations of ATP and the core RNA polymerase are indicated in the figure. At the indicated time points, aliquots of the *in vitro* transcription reactions were spotted on a PEI-cellulose plate (Sigma–Aldrich), which was subsequently developed in 1 M LiCl, 1 M formic acid to remove unincorporated nucleotides. X-ray film was exposed to the resulting TLC plate (top panel), and ^32^P-labeled RNA remaining at the origin of the plate was quantitated (bottom panel). A representative result from two independent experiments is shown. See text for additional details. **B.** Initial rates of non-promoter-specific RNA synthesis by the purified *E. coli* core RNA polymerase in the presence or absence of RapA^R599A/Q602A^ (a RapA mutant lacking transcription-stimulatory and ATP-hydrolytic activities [24]). RapA^R599A/Q602A^ was isolated as previously described [24]. *In vitro* transcription reactions were carried out in buffer B (see Materials and Methods) with dT_20_ (Invitrogen, 0.2 µg/µl); ATP, 1 mM; core RNA polymerase, 250 nM; RapA^R599A/Q602A^, 500 nM. *In vitro* transcription reaction products were analyzed as described in Figure 6A. A representative result of three independent experiments is shown. A schematic for RapA-mediated dissociation of the core enzyme ‘dimers’ is shown at right. *Bottom panels:* Models of a (wild-type) RapA–nucleic acid–ATP complex (15, 24) and RapA^R599A/Q602A^–nucleic acid complex [24]; ssT, single stranded template; dsT, double-stranded template; Q602 (pink) and R599 (magenta) are shown as spheres. **C.** Expression of RapA^R599A/Q602A^ results in a growth defect. M15 *E. coli* cells transformed with pQE32 vector DNA (rectangles, ‘Control A’), plasmid pQE32 Mfd (see Materials and Methods)(circles, ‘Control B’), and plasmid pQE32 RapA^R599A/Q602A^ [24] (triangles) were grown overnight in LB media containing 100 µg/ml ampicillin. Before the experiment, the cultures were diluted (>50-fold) with fresh, pre-warmed to 37°C LB media containing 100 µg/ml ampicillin, and after approximately 75 min protein expression was induced by the addition of IPTG to a final concentration of 0.1 mM. Open and solid symbols represent the results of two independent experiments. Note that the deletion of either *rapA* or *mfd* genes does not lead to growth defects in liquid media [14], [24]; therefore the observed effect of RapA^R599A/Q602A^ is unlikely due to the mutant protein ‘outcompeting’ its wild-type counterpart.

We next sought to study how self-association of the RNA polymerase core enzyme might affect its transcriptional activity. To address this question, we used two independent methods. In the first set of experiments (which took advantage of the relatively high, nanomolar-range DNA-binding affinity of RNA polymerase), we monitored the initial rates of non-promoter-specific RNA synthesis in reactions containing sequentially diluted core enzyme (across its oligomerization range, see Figure 4C) in the presence of excess DNA and nucleoside triphosphate. In the panel shown in Figure 6A, sequential, 5-fold dilutions of the core enzyme were accompanied by concomitant, 5-fold increases in the amount of [α^32^P] ATP in the reactions, so that the expected yields of ^32^P-labeled RNA transcripts would remain unaltered if self-association were to have no effect on the (specific) transcriptional activity of the polymerase. As seen from Figure 6A, the initial rates of RNA synthesis by the core enzyme were boosted at higher core enzyme concentrations (Figure 6A) due to the possible ‘dimerization’ of RNA polymerase. Next, in order to confirm the observed modulation of transcriptional activity of the polymerase during its transition from ‘dimer’ to ‘monomer’, we sought an independent approach, which did not rely on dilutions of the core enzyme. Because (α_2_ββ'ω)_2_ complexes were efficiently dissociated by the RNA polymerase auxiliary SWI/SNF subunit RapA (Figure 2), we used a recently constructed RapA^R599A/Q602A^ mutant with disrupted transcription-stimulatory and ATP-hydrolytic activities [24] as a means to trigger the dissociation of putative tandem transcription complexes at concentrations above K_s_. The R599A/Q602A mutation – located in the vicinity of RapA's putative ATP-binding cleft (Figure 6B, bottom panels) – yields non-functional mutant RapA with folding and solubility properties comparable to those of wild-type RapA [24]. (Note that excess RapA^R599A/Q602A^ does not inhibit the transcriptional activity of the polymerase at low RNA polymerase concentrations [24]). As seen from the data shown in Figure 6B, this independent approach yielded results consistent with the results of the experiments described in Figure 6A. There was a distinct reduction in the initial rate of RNA synthesis in the presence of RapA (Figure 6B, compare lanes A_1_ and B_1_; see also Figure 6B, graph), likely due to RapA-mediated disruption of hypothetical tandem RNA polymerase complexes (Figure 6B, schematic). In accord with this, expression of RapA^R599/Q602^ resulted in a measurable growth defect, arguably due to the dissociation of putative tandem transcription complexes (Figure 6C).

**Figure 7:**
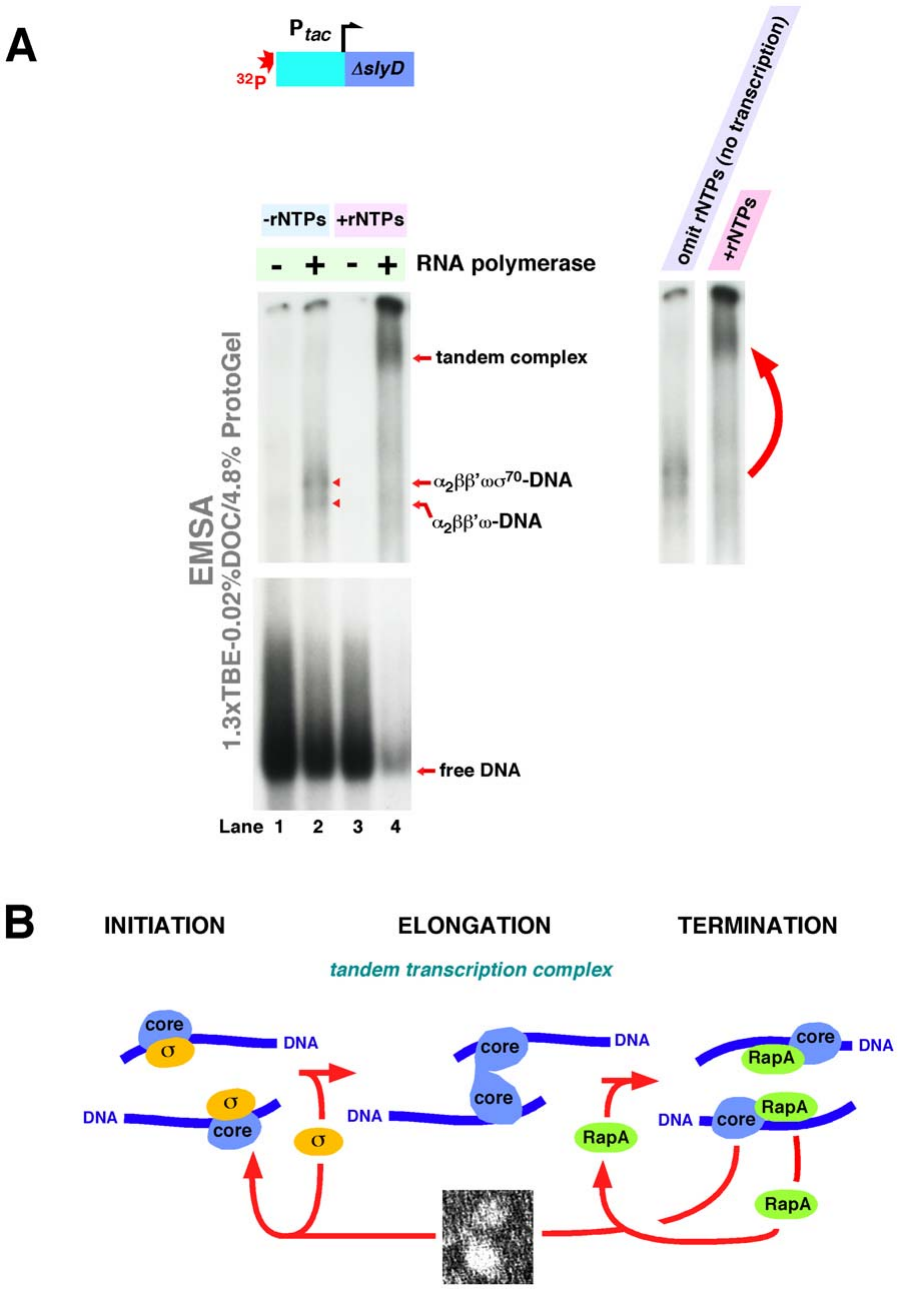
Formation of putative tandem elongation complexes during *in vitro* transcription. Design and purification of DNA templates containing the *tac* and *slyD* promoters [24] are described in Materials and Methods; the templates are also shown schematically at the top in Figures 7**A** **and S2**. The experiments were carried out with RNA polymerase containing approximately 0.5 mol σ^70^/mol core (to mimic the ‘physiological’ ratio of the two forms of RNA polymerase). *In vitro* transcription reactions were carried out in buffer B. When indicated, rNTPs were present at 0.2 mM; purified RNA polymerase, at 16 nM. Following a 3-min incubation at 37°C, the reaction products containing ^32^P-labeled nucleic acids were fractionated by native PAGE; gel components are specified in the figure. **A.** Formation of putative tandem transcription complexes during *in vitro* transcription with a 153-nt DNA template containing the *tac* promoter. Single-copy RNA polymerase–DNA and putative tandem elongation complexes are indicated. Note that the mobility of individual complexes was similar to that seen in the experiments described in Figure 3 (Gels 2 and 3), which included confirmation of the observed protein species/bands after their excision from polyacrylamide gels. Panel at the right: highlight of the results of the experiment. The observed shift in the core RNA polymerase ‘monomer’–tandem equilibrium could be due to rNTP-driven conformational changes in RNA polymerase; stabilization of functional tandems by nascent RNAs also cannot be ruled out. **B.** Schematic for hypothetical oligomerization of transcriptionally active RNA polymerase. See text for details.

We next sought evidence that the elongation (DNA–RNA polymerase–RNA) complexes formed on more conventional DNA templates carrying previously characterized promoters could ‘dimerize’. To this aim, we used two dissimilar model systems/DNA templates, one of which contained the well-characterized *tac* promoter and another the *slyD* gene promoter, which was characterized in our recent work [24]. The *tac* promoter template is shown, schematically, in Figures 7A. In these studies, we gel-fractionated functional transcription complexes in which DNA had been labeled with ^32^P; we then monitored the makeup of the observed complexes in a manner similar to that described in Figures 2 and 3.

We first tested whether the addition of rNTPs could alter the core ‘monomer’–‘dimer’ equilibria. With the ^32^P-labeled dsDNA templates containing the *tac* promoter, the addition of rNTPs shifted the equilibrium in the direction of ‘dimeric’ complexes (Figure 7A, lanes 2 and 4). To prove that the observed effect was not *tac* promoter-specific, we carried out similar *in vitro* transcription studies using DNA templates containing the *slyD* promoter, with largely similar results (Supporting Information, Figure S2).

## Discussion

In this work, we analyzed the oligomeric state(s) of *E. coli* RNA polymerase in the presence or absence of DNA and studied the effect of such oligomerization on the transcriptional activity of the polymerase.

**(a)** We demonstrate that the *E. coli* RNA polymerase core enzyme obtained in a purification procedure based on that described by Hager *et al*. [10] forms stable (α_2_ββ'ω)_2_ complexes. Multiple control experiments indicate that this oligomerization is unlikely due to mediation by RNA polymerase-associated accessory, non-protein molecules. (i) Gel-exclusion chromatography-, (ii) native PAGE-, and (iii) electron microscopy-based analyses yielded evidence supporting the formation of (α_2_ββ'ω)_2_ complexes. The RNA polymerase concentration corresponding to 50% oligomerization (the apparent K_s_, determined under conditions described in Figure 4C) was in the 60–120 nM range. Thus, our results – obtained using three dissimilar techniques – produced no evidence of the formation of higher order core enzyme oligomers reported in early studies. The lack of such higher order oligomers and/or aggregates was particularly apparent during native PAGE-based experiments, in which colloidal Coomassie- and silver-stained gels revealed not even minute traces of such complexes (Figure 3, Gels 2 and 3, lane 1). It is possible that these discrepancies can be explained by the fact that the early studies with *E. coli* RNA polymerase relied primarily on ultracentrifugation, a technique known to be ill-suited for accurate determinations of molecular masses of proteins. This is particularly true for large proteins, whose sedimentation rates could be easily altered by accessory non-protein molecules, such as nucleic acids or lipids.

**(b)** Our work suggests that DNA-bound RNA polymerase may retain the ability to form oligomeric complexes. We show that (α_2_ββ'ω)_2_ complexes can be formed in the presence of transcriptionally active DNA templates. It is clear that if RNA polymerase were to elongate RNA transcripts in tandem *in vivo* – even only under specific conditions – this could have a significant impact on our understanding of the general mechanisms of transcription and its regulation in *Eubacteria*.

**(c)** Importantly, we show that (α_2_ββ'ω)_2_ complexes can be dissociated into single-copy (α_2_ββ'ω) complexes by known protein interactors of the core enzyme, such as RapA, NusA, or σ^70^. We therefore found no evidence for oligomerization of the RNA polymerase holoenzyme under our experimental conditions, contrary to previous reports [8], [21].

**(d)** Our results may explain the efficient separation of the *E. coli* core RNA polymerase and RNA polymerase holoenzyme during Mono Q chromatography. Our data indicate that this separation is likely due to differences in the oligomeric states of the two forms of the polymerase (Figure 3) rather than the presence or absence of σ^70^ alone. Note that our native PAGE-based analyses – in which tandem complexes partially ‘monomerized’ by a detergent (Figure 3A illustrates the detergent-mediated transition of a single species into multiple species with dissimilar electrophoretic mobilities) were further analyzed through the excision of individual bands from gels and identification of their protein content (Figure 3B) – suggested that the significant alterations in mobility which were observed were unlikely to be the result of conformational changes in the core enzyme. Further analyses of the ∼800-kDa core enzyme species by electron microscopy (in solution) and gel-exclusion chromatography allowed us to confirm their identity.

**(e)** We demonstrate that the oligomerization of RNA polymerase may be functionally significant. Our finding that RapA triggers the dissociation of putative tandem complexes likely explains previously reported (but never conclusively explained) observations that RapA, while capable of significant stimulation of multi-round transcription, actually reduces the initial rates of RNA synthesis [12]. It should be noted that other groups working with the *E. coli* RNA polymerase have previously reported the concentration-dependent ‘activation’ of the polymerase. For example, a 2003 study by Epshtein and Nudler reported such a phenomenon [25]. However, the authors attributed the observed effect solely to interactions ‘in *cis*’ (within a transcription complex formed on a single DNA template). Transcriptional activation resulting from the interaction of RNA polymerase with a DNA-binding protein (other than RNA polymerase) is also well known and is, in fact, a basis for the bacterial version of a two-hybrid system. Our work, therefore, points to the possibility that such activation can be achieved through contact with another RNA polymerase molecule transcribing from a different template.

**(f)** Utilizing DNA templates containing either *tac* or *slyD* promoters, we demonstrate ‘dimerization’ of the *E. coli* elongation (DNA–RNA polymerase–nascent RNA) complexes (Figures 7A and S2).

We believe that **(a)–(f)** warrant a hypothesis in which the *E. coli* core RNA polymerase carries out the elongation stage of its catalytic cycle in tandem, while the (sigma-assisted) initiation stage of the transcription cycle is carried out by a single-copy holoenzyme species (Figure 7B). The proposed roles of NusA and RapA in the termination and post-termination stages of the transcription cycle also call for a single-copy RNA polymerase complex; this may reflect the greater complexity and/or decreased processivity of the aforementioned events. None of the data available to us at the moment support a ‘head-to-tail’ stacking of the core enzyme molecules in (α_2_ββ'ω)_2_ complexes. This alternative mode of binding predicts the existence of (2+n) core enzyme complexes (effectively, core enzyme polymers), which, had they been present, should have been apparent during our gel-exclusion chromatography-based and native PAGE-based experiments (see comment ‘**d**’ above). The lack of experimental evidence for ‘head-to-tail’ stacking, therefore, supports the mode of interaction shown schematically in Figures 4–7. Our results support a direct interaction (not mediated by accessory nucleic acids or other non-protein molecules) between individual α_2_ββ'ω complexes. Even though at present we cannot entirely exclude the possibility that the core enzyme obtained in our purification procedure may contain deeply embedded nucleic acid fragments, extensive trials (involving T4 PNK labeling in the presence of [γ^32^P] ATP) carried out under varied experimental conditions consistently failed to detect the presence of exogenous nucleic acids that could contribute to the oligomerization of RNA polymerase. Furthermore, RNase A and DNase I digests of the ‘dimeric’ complexes formed by the core enzyme failed to shift the ‘monomer’–‘dimer’ equilibria (Figure 2). This supports the idea that the complexes in question are stabilized solely or at least predominantly by protein–protein interactions.

It is generally believed that in *Eubacteria* key stages in RNA synthesis are carried out by a single-copy core RNA polymerase complex. While the results presented in this work clearly fall short of proving the existence of tandem RNA polymerase complexes *in vivo*, our data, particularly the *in vitro* transcription experiments, point to the possibility of such an interaction. This hypothesis is also supported by our *in vivo* experiments, in which we show that the expression of RapA^R599/Q602^ (the mutant RapA protein with disrupted transcription-stimulatory and ATP-hydrolytic functions [24]) results in a measurable growth defect (Figure 6C). Arguably, most ‘convincing’ images of transcribing bacterial RNA polymerases were obtained (*in vitro*) with either tethered or surface/grid-bound DNA molecules (thus, under conditions in which the forces involved in DNA–[solid]support interactions almost certainly exceeded those needed for core enzyme dimerization). However, we are not aware of any study in which analysis of the oligomeric states of the DNA-bound polymerase was carried out in a ‘soluble’ system. If *E. coli* RNA polymerase is indeed able to synthesize RNA and track DNA in tandem, this could potentially open new insights into the biophysics of transcription. It is possible to speculate that the energy needed for translocation of the polymerase along DNA could be at least partially derived from conformational changes of the nucleoid. (Thus, the energy generated when DNA strands facing each other slide in the opposite direction could be ‘recycled’). Discrepancies between the *in vivo* and *in vitro* transcription elongation rates may indirectly support this hypothesis. We also speculate that transcription by tandem RNA polymerase complexes initiated at hypothetical bidirectional ‘origins of transcription’ may explain recurring switches of the direction of transcription in bacterial genomes.

## Materials and Methods

### Enzymes

*E. coli* RNA polymerase was purified as described [20], [24]; a schematic for the purification procedure is shown in Figure 1A. This procedure was derived from the protocol originally described by Hager *et al.* [10]. RapA and NusA were purified as described [13], [20], [22].

*Gel-filtration-based* studies were carried out using a Superose 6 10/300 column (GE Healthcare) integrated into an AKTA FPLC system (GE Healthcare). Samples were injected manually; the typical sample volume was 40 µl. Chromatography was carried out in 0.01 M Tris–HCl (pH 7.5), 5% glycerol, 0.2 mM EDTA (TGED buffer) containing 100 mM NaCl, at a flow rate of 0.5 ml/min. Elution times of the protein peaks were determined using Unicorn software (version 5.01, GE Healthcare). The core RNA polymerase utilized in this set of experiments (samples A1–A3) was isolated as described [24]; sample B1, as described [20]. The RNA polymerase holoenzyme was isolated as previously described [24].

*Electrophoretic Mobility Shift Assays* (EMSA) were carried out using a Gibco BRL SA-type vertical electrophoresis apparatus. Polyacrylamide gels were cast using 30% Protogel (National Diagnostics); 10× TBE (KD Medical) stock was diluted tenfold for the gel mix; 0.5× TBE was used as a running buffer. Unless indicated otherwise in Figure Legends, EMSA gels contained no detergents or chaotropic agents. Purified DNA probes were end-labeled using T4 polynucleotide kinase (Sigma) and [γ^32^P] ATP (MP Biomedicals). ^32^P 5′ end-labeling and gel-purification of nucleic acid probes was carried out as previously described [13]. Typically, each 10-µl binding reaction contained 1 µl of 10× reaction buffer D (10× reaction buffer D: 200 mM Tris-acetate, 100 mM magnesium acetate, 500 mM potassium acetate, 10 mM dithiothreitol, pH 7.8), ∼2000 cpm of ^32^P-labeled DNA, and purified proteins; protein concentrations are specified in Figure Legends.

*Electron microscopic experiments* were carried out essentially as previously described [23]. Core RNA polymerase samples (0.05–0.2 mg/ml in 1× TGED buffer containing 100 mM NaCl) were negatively stained with 2% phosphotungstic acid at pH 7.0. Measurements were typically taken at a magnification of 75,000×.

*In vitro transcription* experiments were carried out as previously described [13] in either 50 mM Tris–HCl (pH 7.8), 10 mM MgCl_2_, 100 mM NaCl, 1 mM dithiothreitol (buffer B) or 1× buffer D. Synthetic DNA templates were obtained from Invitrogen. DNAs were purified either by cartridge or gel extraction (Qiagen). In kinetic experiments, enzyme–DNA mixtures were pre-incubated at room temperature for 15 min, and the transcription reaction was initiated by the addition of rNTPs.

*DNA templates* containing *tac* and *slyD* promoters were constructed as follows. The 153-nt DNA containing the *tac* promoter was constructed as previously described [24]. In brief, linear DNA was generated by PCR from MG1655 *E. coli* chromosomal DNA using DNA primers MS722 (5′-AATTCTGTTGACAATTAATCATCGGCTCGTATAATGTGGGAATTGTGAGCGGATAACAATTTCACACAGGAAACACGCGAAGCGACTGAAGAAGAAC) (this primer introduced the *tac* promoter into the amplified DNA) and MS731 (5′-GTCGTGGTCGTGATCGTGGTGGTGATC) (thus, the truncated *slyD* sequence downstream from *tac* was further shortened to 78 nucleotides to generate a runoff transcript during *in vitro* transcription; note that the transcriptional activity of a similar DNA construct was confirmed [24]). Amplified DNA was gel-purified using the Qiagen Gel Extraction kit (Cat. No. 28704). The 965-nt linear DNA template containing the *slyD* operon was amplified from MG1655 *E. coli* chromosomal DNA using MS696 and MS697 DNA primers, as previously described [24]. The amplified DNA was purified by FPLC, as previously described [24]. The transcriptional activity of this DNA template and the positions of the *slyD* promoter and terminator were also independently verified [24]. The purified DNAs were end-labeled using T4 PNK and [γ^32^P]ATP (MP Biomedicals); end-labeling and gel-purification of ^32^P-labeled DNAs were carried out as previously described [13].

### *In vivo* experiments

Plasmid pQE32Mfd used in the experiments described in Figure 6C was constructed as follows. The *mfd* gene was amplified from MG1655 *E. coli* chromosomal DNA using MS781 (5′-GCCTATCCCGGGGGATCCTCATGCCTGAACAATATCGTTATACGCTGCCC) and MS782 (5′-GCCAAACCCGGGAAGCTTTTATTACCATTAAGCGATCGCGTTCTCTTCCAG) DNA primers and the Expand High Fidelity^PLUS^ PCR kit (Roche Diagnostics, Cat. No. 03-300-226-001); reactions were conducted as suggested by the manufacturer. Amplified linear DNA was gel-purified using the Qiagen Gel Extraction kit, digested with BamHI/HindIII and ligated into pQE32 vector DNA (Qiagen) digested with BamHI/HindIII (BamHI, HindIII, and T4 DNA Ligase were obtained from New England Biolabs). Expression of a full-length protein from the resulting construct was confirmed by SDS-PAGE.

## Supporting Information

Figure S1 Disruption of the putative tandem complexes formed by the *E. coli* RNA polymerase in the presence of NusA. Reactions were similar to those in Figure 2D, except that samples contained no nucleic acids and the amount of the core RNA polymerase in each 20-µl reaction was increased approximately 5-fold in order to visualize the protein complexes by Coomassie staining.
(TIF)

Figure S2 Formation of putative tandem elongation complexes during *in vitro* transcription. Design and purification of the 965-nt linear DNA template containing the *slyD* promoter [24] is described in Materials and Methods; the template is also shown schematically at the top. The experiment was carried out in buffer D, using RNA polymerase containing approximately 0.5 mol σ^70^/mol core (to mimic the ‘physiological‘ ratio of the two forms of RNA polymerase). When indicated, rNTPs were present at 0.2 mM. Following a 3-min incubation at 37°C, the reaction products containing ^32^P-labeled nucleic acids were fractionated by agarose gel electrophoresis. Purified RNA polymerase was present at either 15.9 nM (lanes 2 and 4) or 79.6 nM (lanes 6 and 8). Single-copy RNA polymerase-DNA (M) and putative tandem elongation complexes (D) are indicated. Panel at the left: the purified ^32^P-labeled DNA probe is shown on an ethidium bromide-stained 1% agarose gel next to a 1-kb DNA ladder (New England Biolabs).
(TIF)
