# Supplementary figures and images for: Oligomerization of the E. coli Core RNA Polymerase: Formation of (α2ββ'ω)2–DNA Complexes and Regulation of the Oligomerization by Auxiliary Subunits
Source: PLoS One. 2011 Apr 20;6(4):e18990. doi: 10.1371/journal.pone.0018990 (PMC3080401; doi:10.1371/journal.pone.0018990)

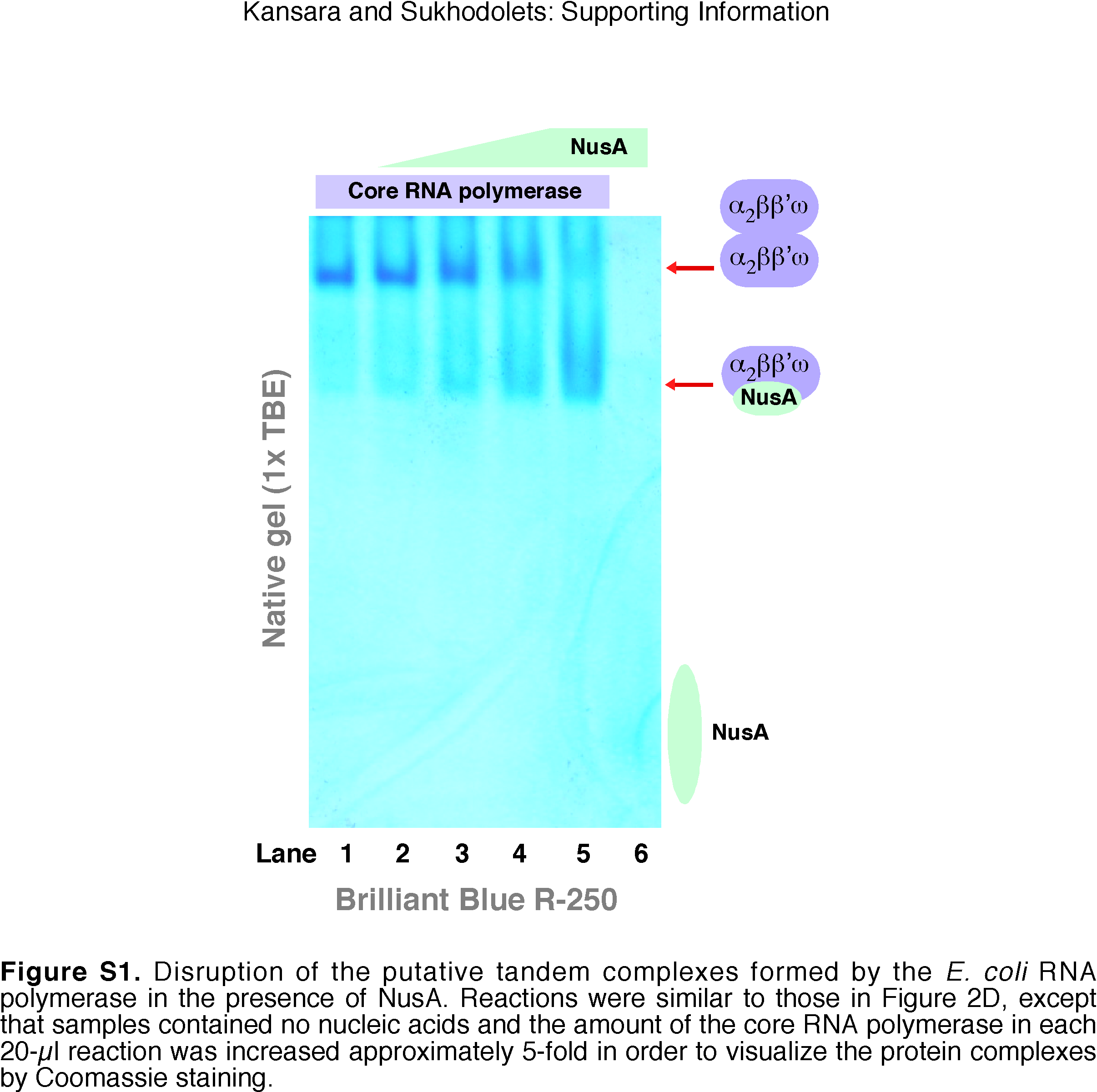

Supplement: Figure S1 — Disruption of the putative tandem complexes formed by the E. coli RNA polymerase in the presence of NusA. Reactions were similar to those in Figure 2D, except that samples contained no nucleic acids and the amount of the core RNA polymerase in each 20-µl reaction was increased approximately 5-fold in order to visualize the protein complexes by Coomassie staining. (TIF) [file pone.0018990.s001.tif]

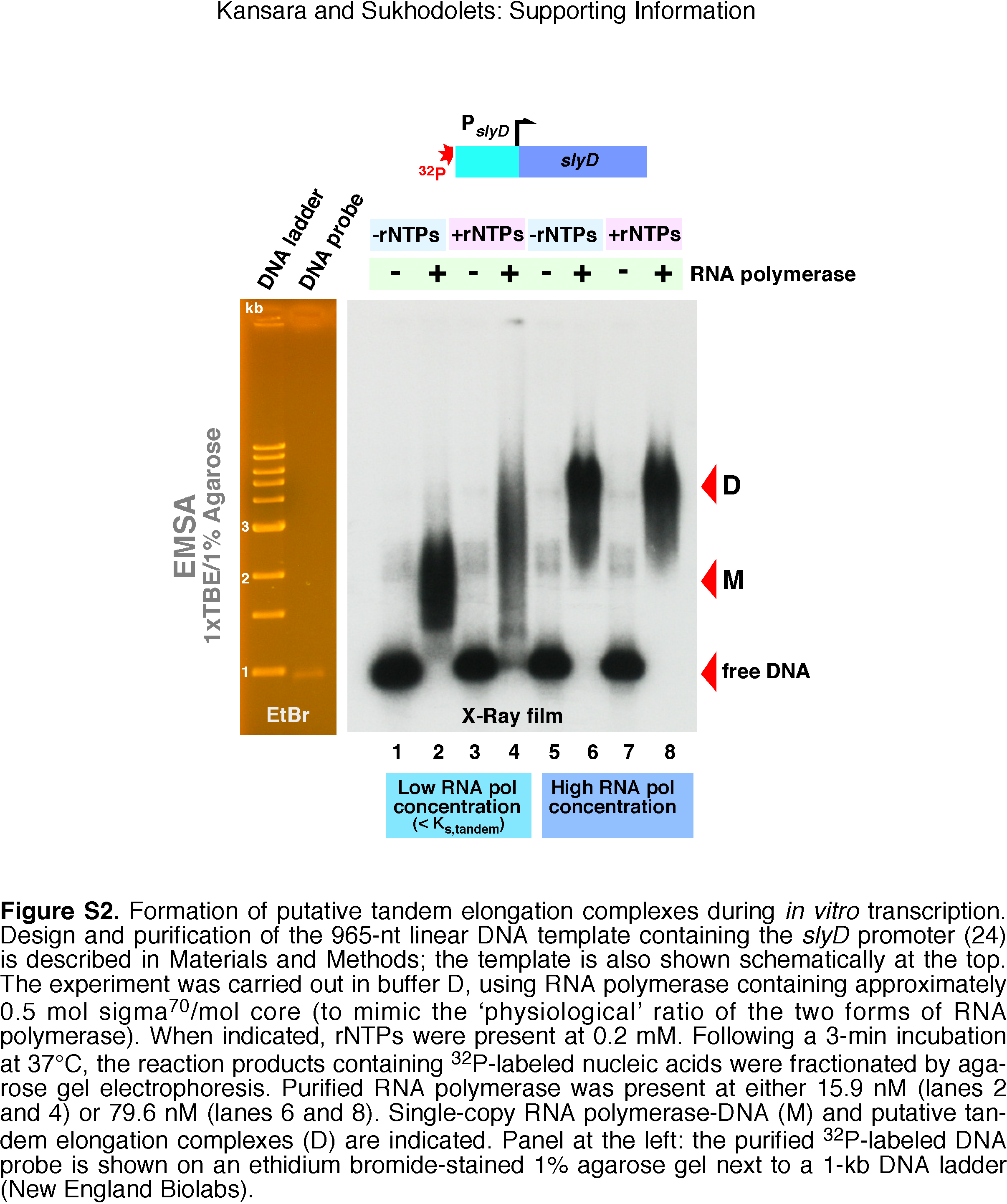

Supplement: Figure S2 — Formation of putative tandem elongation complexes during in vitro transcription. Design and purification of the 965-nt linear DNA template containing the slyD promoter [24] is described in Materials and Methods; the template is also shown schematically at the top. The experiment was carried out in buffer D, using RNA polymerase containing approximately 0.5 mol σ70/mol core (to mimic the ‘physiological‘ ratio of the two forms of RNA polymerase). When indicated, rNTPs were present at 0.2 mM. Following a 3-min incubation at 37°C, the reaction products containing 32P-labeled nucleic acids were fractionated by agarose gel electrophoresis. Purified RNA polymerase was present at either 15.9 nM (lanes 2 and 4) or 79.6 nM (lanes 6 and 8). Single-copy RNA polymerase-DNA (M) and putative tandem elongation complexes (D) are indicated. Panel at the left: the purified 32P-labeled DNA probe is shown on an ethidium bromide-stained 1% agarose gel next to a 1-kb DNA ladder (New England Biolabs). (TIF) [file pone.0018990.s002.tif]
